# Quantifying the impact of vaccination on transmission and diversity of influenza A variants in pigs

**DOI:** 10.1128/jvi.01245-24

**Published:** 2024-11-12

**Authors:** Chong Li, Marie R. Culhane, Declan C. Schroeder, Maxim C-J. Cheeran, Lucina Galina Pantoja, Micah L. Jansen, Montserrat Torremorell

**Affiliations:** 1 College of Veterinary Medicine, University of Minnesota, St. Paul, Minnesota, USA; 2 Zoetis, Parsippany, New Jersey, USA; Cornell University Baker Institute for Animal Health, Ithaca, New York, USA

**Keywords:** influenza A virus, variant transmission, genetic diversity, pig, vaccination

## Abstract

Global evolutionary dynamics of influenza A virus (IAV) are fundamentally driven by the extent of virus diversity generated, transmitted, and shaped in individual hosts. How vaccination affects the degree of IAV genetic diversity that can be transmitted and expanded in pigs is unknown. To evaluate the effect of vaccination on the transmission of genetically distinct IAV variants and their diversity after transmission in pigs, we examined the whole genome of IAV recovered from the nasal cavities of pigs vaccinated with different influenza immunization regimens after being infected simultaneously by H1N1 and H3N2 IAVs using a seeder pig model. We found that the seeder pigs harbored more diversified virus populations than the contact pigs. Among contact pigs, H3N2 and H1N1 viruses recovered from pigs vaccinated with a single dose of an unmatched modified live vaccine generally accumulated more extensive genetic mutations than non-vaccinated pigs. Furthermore, the non-sterilizing immunity elicited by the single-dose-modified live vaccine may have exerted positive selection on H1 antigenic regions as we detected significantly higher nonsynonymous but lower synonymous evolutionary rates in H1 antigenic regions than non-antigenic regions. In addition, we observed that the vaccinated pigs shared significantly less proportion of H3N2 variants with seeder pigs than unvaccinated pigs. These results indicated that vaccination might reduce the impact of transmitted influenza variants on the overall diversity of IAV populations harbored in recipient pigs and that within-host genetic selection of IAV is more likely to occur in pigs vaccinated with improperly matched vaccines.

**IMPORTANCE:**

Understanding how vaccination shapes the diversity of influenza variants that transmit and propagate among pigs is essential for designing effective IAV surveillance and control programs. Current knowledge about the transmission of IAV variants has primarily been explored in humans during natural infection. However, how immunity elicited by improperly matched vaccines affects the degree of IAV genetic diversity that can be transmitted and expanded in pigs at the whole-genome level is unknown. We analyzed IAV sequences from samples collected daily from experimentally infected pigs vaccinated with various protocols in a field-represented IAV co-infection model. We found that vaccine-induced non-sterilizing immunity might promote genetic variation on the IAV genome and drive positive selection at antigenic sites during infection. In addition, a smaller proportion of H3N2 viral variants were shared between seeder pigs and vaccinated pigs, suggesting the influence of vaccination on shaping the virus genomic diversity in recipient pigs during the transmission events.

## INTRODUCTION

Influenza A virus (IAV) infections are costly to pig producers and a risk to public health. Enhanced control of swine influenza should help alleviate pig productivity losses, improve animal well-being (1), and benefit public health since pigs can be a source of novel viruses with zoonotic and pandemic potential (2). Effective control strategies are urgently needed to mitigate the burden of IAV infections in pigs and decrease the occurrence of virus spillover events at the swine-human interface (3).

The high mutation rates and fast-expanding nature of IAV result in a swarm of genetically closely related progeny virus variants during each production cycle, resulting in heterogeneous viral populations within the hosts, also known as quasispecies (4, 5). The error-prone replication of the RNA polymerase, together with the swap of gene segments between distinct IAV viruses (reassortment), facilitates the rapid accumulation of genetic diversity, promoting virus evolution and generating novel variants. Pigs are an intermediate host for IAV infections and play an important role in influenza ecology as they are susceptible to IAVs from multiple hosts (6). Since 1998, abundant viruses with diverse subtypes and genotypes that contain genetic material originating from swine, human, and avian IAVs have been identified in North American pigs, which makes IAV infections extremely difficult to control (7, 8). Thus, minimizing IAV diversity in pigs is critical to control IAV effectively.

Influenza vaccination is commonly used to mitigate the disease burden in pigs, and its usage is common in US pig farms (9). Despite the clinical benefit of using IAV vaccination to reduce disease severity and limit virus infection, the high genetic diversity of IAV found in pigs still enables IAV to replicate, evolve, and transmit among vaccinated animals (10, 11). Most of the licensed IAV vaccines used in the swine industry are multivalent and contain whole-cell inactivated viruses, or replicon particles (12–14). A live attenuated vaccine available at the time of this study was temporarily available in the US market but is no longer available commercially (15). In general, the vaccine’s protective efficacy is compromised due to the emergence of immune escape mutants and reassortant viruses, the co-circulation of antigenically distinct virus variants, and the limited cross-protection offered by commercially available vaccines (16).

The influenza hemagglutinin (HA) and neuraminidase (NA) proteins determine the virus antigenic subtype. HA is the primary target for neutralizing immunity, which is undoubtedly important for influenza surveillance and vaccine design (17). However, the humoral immunity raised by most of the licensed IAV vaccines is against the whole virus, and host immunity exerts selective pressure through the whole genome of the IAV variant populations (18). Given the fact that pigs of different immune and infection statuses co-exist in farms, the viruses may go through frequent population bottlenecks and encounter different selective host environments (19, 20). As a result, viral populations accumulate mutations at the whole-genome level to maximize their possibility of replicating under repeated bottlenecks (21). Whether the variants can be successfully selected in infected hosts and disseminated in pig populations depends on the genetic features and functional coordination among the whole virus components rather than just antigenicity (22, 23).

The development of high-throughput sequencing technologies has provided a feasible and powerful approach to measure the genetic heterogeneity of IAV populations. Despite multiple studies that have assessed the transmission of viral variants during natural infections and the impact of vaccination on IAV within-host diversity, little knowledge revealed how vaccination affects the viral variant transmission between individual hosts. In humans, studies have shown that within-host IAV diversity is dominated by low-frequency variants, which are mainly shaped by purifying selection and genetic drift (24–27). In addition, the tight transmission bottlenecks were observed to constrain the transmission of low-frequency IAV variants in multiple transmission models (24). Although vaccination has been associated with a positive selection of IAV antigenic proteins at the global scale (28, 29), a study showed that seasonal influenza vaccination did not exhibit significant changes in H3N2 diversity at the intra-host level during infections (27). Compared with human studies, the knowledge of the impact of vaccination on the transmission of influenza variants and its impact on variant diversity between donor and recipient pigs is limited and mainly derived from the analysis of HA1 sequences using a serial IAV transmission model (30, 31). These studies found that the immune response could potentially drive the time-dependent positive and negative selection of specific HA alleles, but vaccination did not appear to have a major effect on the size of the transmission bottleneck among pigs (30, 31). Whether similar findings can be found in a swine co-infection model, to what degree genetic diversity can be transmitted, and how the transmitted influenza variants impact the virus within-host diversity among pigs with different immune statuses, based on the analysis of the whole genome of IAV at multiple time points throughout infection, is unknown.

In this study, we assessed and quantified the transmission of genetically distinct IAV variants and the within-host diversity of these variants in pigs vaccinated with whole inactivated vaccines (WIV) or a live attenuated influenza vaccine (LAIV) by evaluating the whole-genome of IAV recovered from nasal swabs collected throughout infection (32). We used a room-based seeder pig co-infection model to simultaneously infect pigs of different immune statuses. The nasal swabs from seeder pigs enabled us to control the background genomic variation of source viruses with relatively high confidence and helped us better understand the extent of transmission of IAV variants and virus genetic variation due to vaccination. Here, we refer to “variant” as the genetically distinct IAV mutants detected through whole-genome sequencing after comparison to the source viruses shed from seeder pigs at the beginning of the study. The results of this study will strengthen the knowledge regarding the evolutionary strategy of IAV variants to transmit and expand in pigs under immune pressure from vaccination. This, in turn, will help develop more effective influenza control programs to benefit both swine production and human public health.

## RESULTS

### Background information of study subjects and sample sequencing

We characterized the IAV variants from the upper respiratory tract, specifically the nasal cavity, of pigs vaccinated and challenged with two subtypes of IAV, an H1N1 and an H3N2 using a seeder pig model (32). The detailed information on the challenge model and protective efficacy of the different vaccination protocols has been illustrated in Material and Methods (32). In addition to the H1N1 and H3N2 seeder pigs, we classified the contact pigs based on the number of vaccine doses administrated as PRIME BOOST, in which pigs received two doses of vaccines; SINGLE LAIV, in which pigs received a single dose of a live attenuated vaccine, and NO VAC (non-vaccinated) pigs.

**Fig 1:**
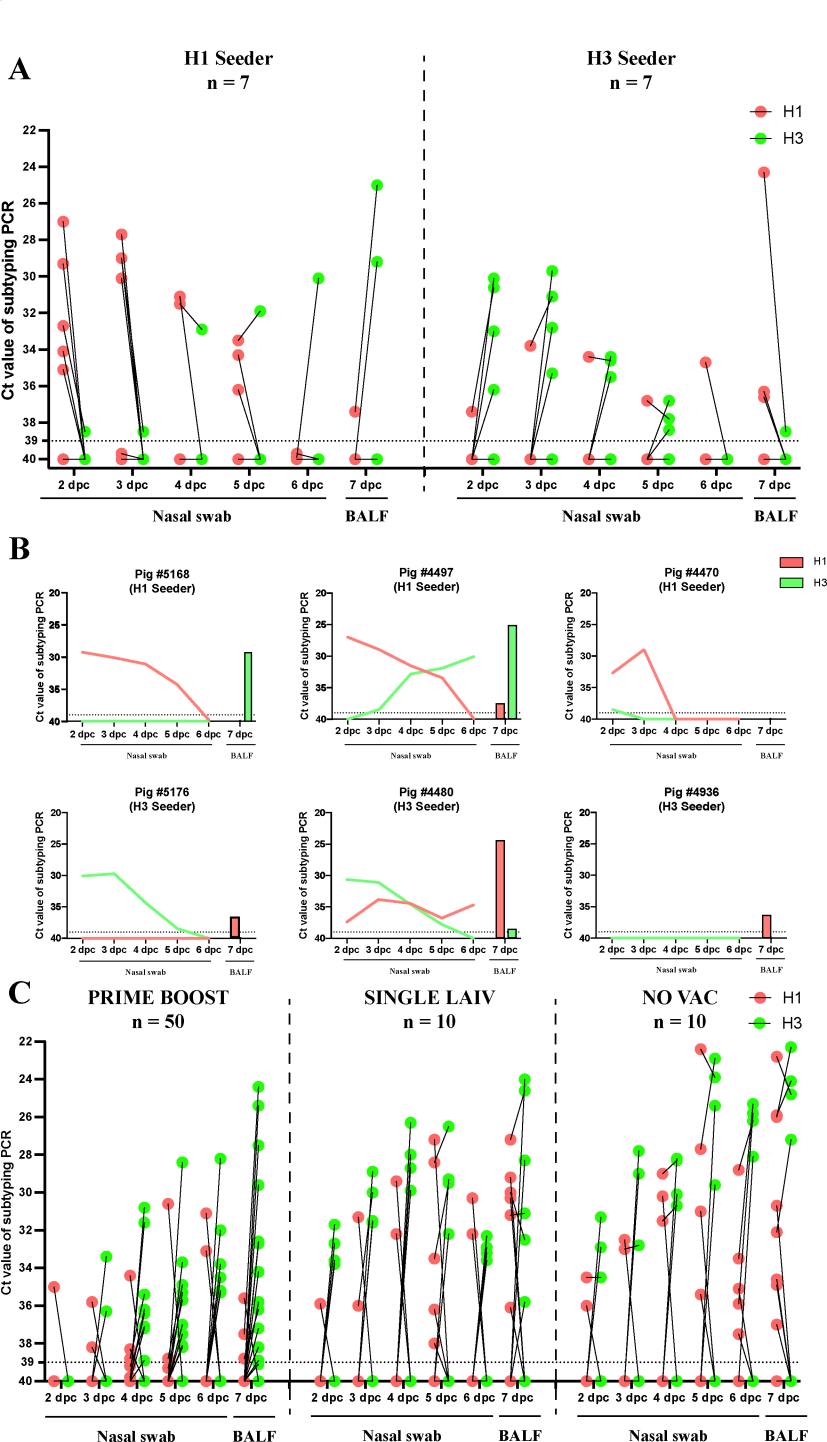
Shedding dynamics of influenza A virus (IAV) in inoculated and contact pigs after commingling them together. (**A**) The shedding dynamics of H1 and H3 IAVs in the nasal cavities (2–6 dpc) and lungs (7 dpc) in inoculated pigs (H1 and H3 seeder pigs). The red and light green data points represent the Ct values of each sample’s H1 and H3 subtyping rRT-PCR, respectively. The short line connecting the red and green dots from the same time point indicates the Ct values of H1 and H3 genes from the same sample collected in pigs on the corresponding day. The dashed line displayed the positive Ct threshold, which is 39. (**B**) Shedding pattern of IAV in seeder pigs confirmed with co-infections caused by H1 and H3 IAV subtypes. The red and light green lines represent the Ct values of the H1 and H3 subtypes from each pig’s nasal swabs (2–6 dpc), respectively. The bars represent the Ct values of the BALF samples at necropsy (7 dpc) with the same color setting. The dashed line indicates the positive threshold of Ct value (Ct = 39) for IAV subtyping rRT-PCR which targets the H1 and H3 genes. (**C**) The shedding dynamics of H1 and H3 IAV viruses in contact pigs (PRIME BOOST, SINGLE LAIV, and NO VAC pigs) was tested by HA subtyping rRT-PCR. The figure setting is same as the figure panel A.

Here, PRIME BOOST pigs had significantly higher average HI titers against both challenge viruses compared to NO VAC pigs and SINGLE LAIV pigs (Table S1). The contact pigs in three groups had a close number of H3-specific interferon (IFN)-γ-secreting cell counts measured by ELISPOT. A higher number of H1-specific IFN-γ cell counts was observed in NO VAC pigs compared to PRIME BOOST and SINGLE LAIV pigs (Table S1). To assess the extent of H1N1 and H3N2 virus shedding for the seeder and contact pigs after commingling them together, we performed HA subtyping rRT-PCR from nasal swabs collected daily from 2 to 6 days post-contact (dpc) and bronchoalveolar lavage fluid (BALF) samples at necropsy (7 dpc). During the 7-day observation, both H1 and H3 seeder pigs exhibited declining viral shedding in nasal cavities of the primary inoculated virus followed by one or two pigs shedding the virus of the other subtype (Fig. 1A). At necropsy, for both H1 and H3 seeders, more pigs shed the virus that transmitted from the other seeder pig in the same room than the primary inoculated virus. At the individual pig level, we found that 6 out of 14 (43%) seeder pigs had co-infections given that the subtype detected in the lungs or nasal cavities was different from the one of the inoculations. Among these pigs, five pigs had apparently cleared their primary IAV infection and at necropsy, they had mainly the subtype of the other seeder pig (Fig. 1B). Among contact pigs, pigs shed more H3N2 viruses, as detected in their nasal cavities and lungs, than the H1N1 viruses from pigs regardless of vaccination status (Fig. 1C). The PRIME BOOST pigs generally shed fewer viruses than SINGLE LAIV and NO VAC pigs for both H1N1 and H3N2 viruses throughout the study, and there were statistical differences on average Ct values in PRIME BOOST pigs compared with NO VAC and/or SINGLE LAIV pigs for H1 and H3 subtypes at certain times (Table S2). Similar amounts of H1N1 and H3N2 viruses were shed from SINGLE LAIV and NO VAC pigs at all sampling times (Table S2). In addition, we observed fewer number of PRIME BOOST pigs shedding both H1N1 and H3N2 viruses than NO VAC and SINGLE LAIV pigs at most of the sampling times. Moreover, we only detected co-infection in 4 out of 50 (8%) PRIME BOOST pigs during the whole observation period, which is far less than the co-infection levels detected from the other two groups (vs SINGLE LAIV – 5/10 (50%), *P* < 0.001; vs NO VAC – 4/10 (40%), *P* = 0.007, Chi-Square test).

We successfully sequenced 23 H1N1 nasal swabs and 45 H3N2 nasal swabs from contact pigs in three groups. In addition, we sequenced the H1N1 genomes of 11 nasal swabs from H1 seeders and H3N2 genomes of 6 nasal swabs from H3 seeders, which served as the background reference for H1N1 and H3N2 genome analysis, respectively (Table 1). We did not detect significant differences in average Ct values (H1N1: *P* = 0.579, H3N2: *P* = 0.047, Kruskal-Wallis rank sum test; No significant difference detected in pairwise comparisons by Dunn’s test) and sequencing coverage (H1N1: *P* = 0.457, H3N2: *P* = 0.894, Kruskal-Wallis rank sum test) in sequenced samples from different groups for both viruses.

**TABLE 1:** Samples analyzed from a co-infection model using seeder pigs inoculated with either an H1N1 or an H3N2 influenza A virus

Virus	Category	Treatment	Total number of sequenced samples	Total number of samples with fully amplified genomes	Total consensus nucleotides	Total identified SNV (Nonsyn/Syn/Stop)^*c*^	Average mutation rate (SD)^*d*^	Average Ct value (SD)^*b*^	Mean coverage (SD)^*a*^
H1N1	Contact pig	PRIME BOOST	4	4	52,532	71 (41/30/0)	0.00126 (0.00025)	27.10 (2.55)	8,650 (1,656)
		SINGLE LAIV	9	8	115,923	146 (87/58/1)	0.00121 (0.00069)	25.52 (3.14)	6,607 (3,833)
		NO VAC	10	9	128,219	234 (144/84/6)	0.00170 (0.00130)	23.54 (4.98)	6,648 (3,145)
	Seeder pig	H1N1 inoculate	11	11	144,463	361 (197/158/6)	0.00226 (0.00079)	25.74 (3.31）	8,762 (3,141）
H3N2	Contact pig	PRIME BOOST	8	8	105,064	163 (86/73/4)	0.00141^AB^ (0.00089)	26.53 (3.69)	8,008 (843)
		SINGLE LAIV	19	18	244,264	404 (227/170/7)	0.00148^AB^ (0.00112)	24.93 (3.46)	7,303 (2,693)
		NO VAC	18	18	236,394	209 (102/105/2)	0.00082^A^ (0.00055)	22.65 (4.33)	8,233 (3,301)
	Seeder pig	H3N2 inoculate	6	6	78,798	214 (124/87/3)	0.00255^B^ (0.00117)	27.27 (2.71)	7,313 (3,116)

^*a*^ The genome coverage of each sample was computed as the average depth of gene reads overlapped on the whole influenza (IAV) consensus genomes.

^*b*^ Ct: cycle threshold, SD: standard deviation. The sample Ct values were measured by influenza Matrix real-time PCR.

^*c*^ The column displayed the total number of identified single nucleotide variants (SNV) and the number of nonsynonymous (Nonsyn), synonymous (Syn), and stop-gained (Stop) SNVs detected in pigs from each group.

^*d*^ The mutation rates were measured as the number of segregating sites per consensus nucleotide. The mean mutation rates from seeder and contact pigs by treatment groups were compared for each virus by the Kruskal-Wallis rank-sum test followed by Dunn’s test. The *P*-values were corrected by the Benjamini-Hochberg method. Statistical differences (*P* < 0.05) are displayed in bold font with different superscripts.

### Influenza variants identified in seeder and contact pigs

All the detected H1N1 and H3N2 single nucleotide variants (SNV) that met the inclusion criteria of SNV identification are reported in native H1 and H3 numbering schemes (including the signal peptide), respectively. We detected a total of 451 H1N1 SNVs and 776 H3N2 SNVs from contact pigs. In addition, we identified 361 H1N1 and 214 H3N2 SNVs in H1 and H3 seeders, respectively, which are summarized in Table 1. We did not observe significant differences among contact pigs on the number of nucleotide mutations per sample after normalizing by the length of sequenced nucleotides for both H1N1 and H3N2 viruses. However, we found a higher average number of mutations per nucleotide site in samples of H3N2 virus from H3 seeders than NO VAC pigs (*P* = 0.007) (Table 1).

**Fig 2:**
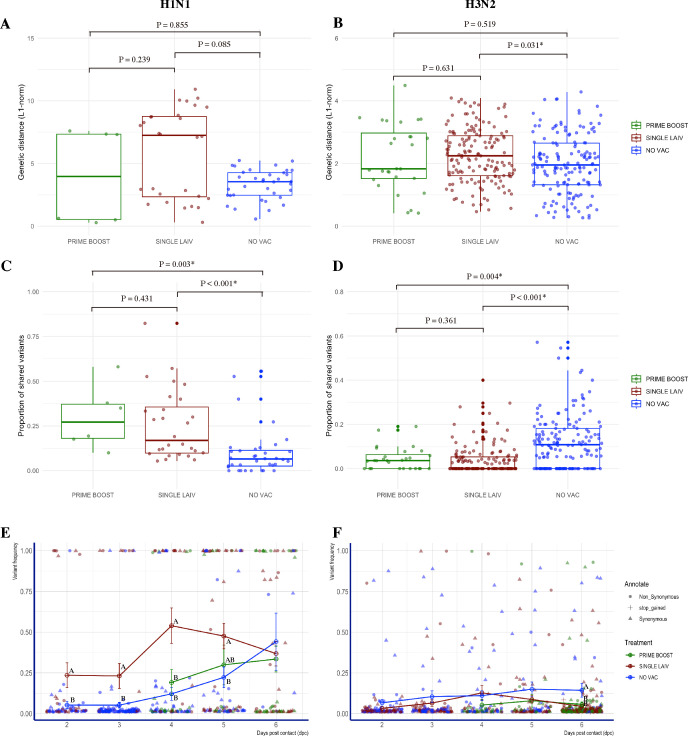
Characterization of within-host diversity of H1N1 and H3N2 viruses in pigs from different treatment groups. Pairwise genetic distance in L1-norm of H1N1 (**A**) and H3N2 (**B**) viruses from contact pigs in different treatment groups. The boxplots display the distribution of values of overall genetic distance in L1-norm for all possible sample pairs in each treatment group, and each dot represents a unique sample combination. Pairwise shared variant proportions on H1N1 (**C**) and H3N2 (**D**) viruses in contact pigs from different treatment groups. The boxplots display the distribution of values of the proportions of shared variants for all possible sample pairs in each treatment group, and each dot represents a unique sample combination. The average L1-norm genetic distance values or the average shared variant proportions values of sample pairs from different treatment groups were compared by the Kruskal-Wallis rank-sum test, followed by Dunn’s test for multiple group comparisons with the Benjamini-Hochberg method for *P*-value adjustment. The samples that failed to amplify the complete eight segments of the IAV genome were discarded from the analysis. The temporal dynamics of variant frequencies in H1N1 (**E**) and H3N2 (**F**) virus populations in pigs from various groups. All the identified SNVs from the sequenced samples collected in PRIME BOOST (green), SINGLE LAIV (brown), and NO VAC (blue) pigs are displayed by days post-contact. The lines include mean and standard error (SE) values of the frequencies of viral variants detected in pigs from different groups on each day compared by student t-test (at days 2 and 3 dpc) and one-way ANOVA (at days 4, 5, and 6 dpc) with Tukey’s HSD for multiple pairwise comparisons.

To measure the similarity of the IAV populations within each treatment group, we conducted all versus all pairwise comparisons across the data set. We calculated the L1-norm genetic distance and the proportion of shared variants at the whole-genome level for each two-sample combination from the same group after excluding the samples for which the whole genome was not amplified. The pairwise comparisons were then grouped based on the origins of each sample. Both subtypes of viruses within SINGLE LAIV pigs displayed the highest genetic distance among contact pigs from the three treatment groups. However, we only detected a significant difference in H3N2 IAVs between SINGLE LAIV and NO VAC pigs (Fig. 2A and B). When comparing the proportion of variants shared between samples from each group, the lowest proportion of variants were shared between NO VAC samples versus PRIME BOOST and SINGLE LAIV samples on H1N1 virus (Fig. 2C). By contrast, NO VAC samples shared higher proportions of H3N2 variants compared to PRIME BOOST and SINGLE LAIV samples (Fig. 2D).

On average, the frequencies of H1N1 SNVs detected in contact pigs were significantly higher than those of H3N2 (*P* < 0.001, unpaired t-test). About 74.7% (337/451) of H1N1 and 88.0% (683/776) of H3N2 SNVs had variant frequencies of less or equal to 10%, and H1N1 (89/451) had more variants than H3N2 (33/776) at consensus level with frequencies ranging from 50% to 100% (*P* < 0.001, Chi-square test). We detected higher mean variant frequencies of H1N1 SNVs in SINGLE LAIV pigs than in NO VAC pigs at 2 to 5 dpc, and larger average H1N1 variant frequency observed in SINGLE LAIV pigs than PRIME BOOST pigs at 4 dpc (Fig. 2E). There was similar variant frequency observed on H3N2 SNVs among treatment groups across all study time-points, except for NO VAC pigs that had higher mean variant frequency of H3N2 SNVs than SINGLE LAIV and PRIME BOOST pigs at 6 dpc (Fig. 2F).

### Similar patterns of IAV within-host diversity in seeder and contact pigs

**Fig 3:**
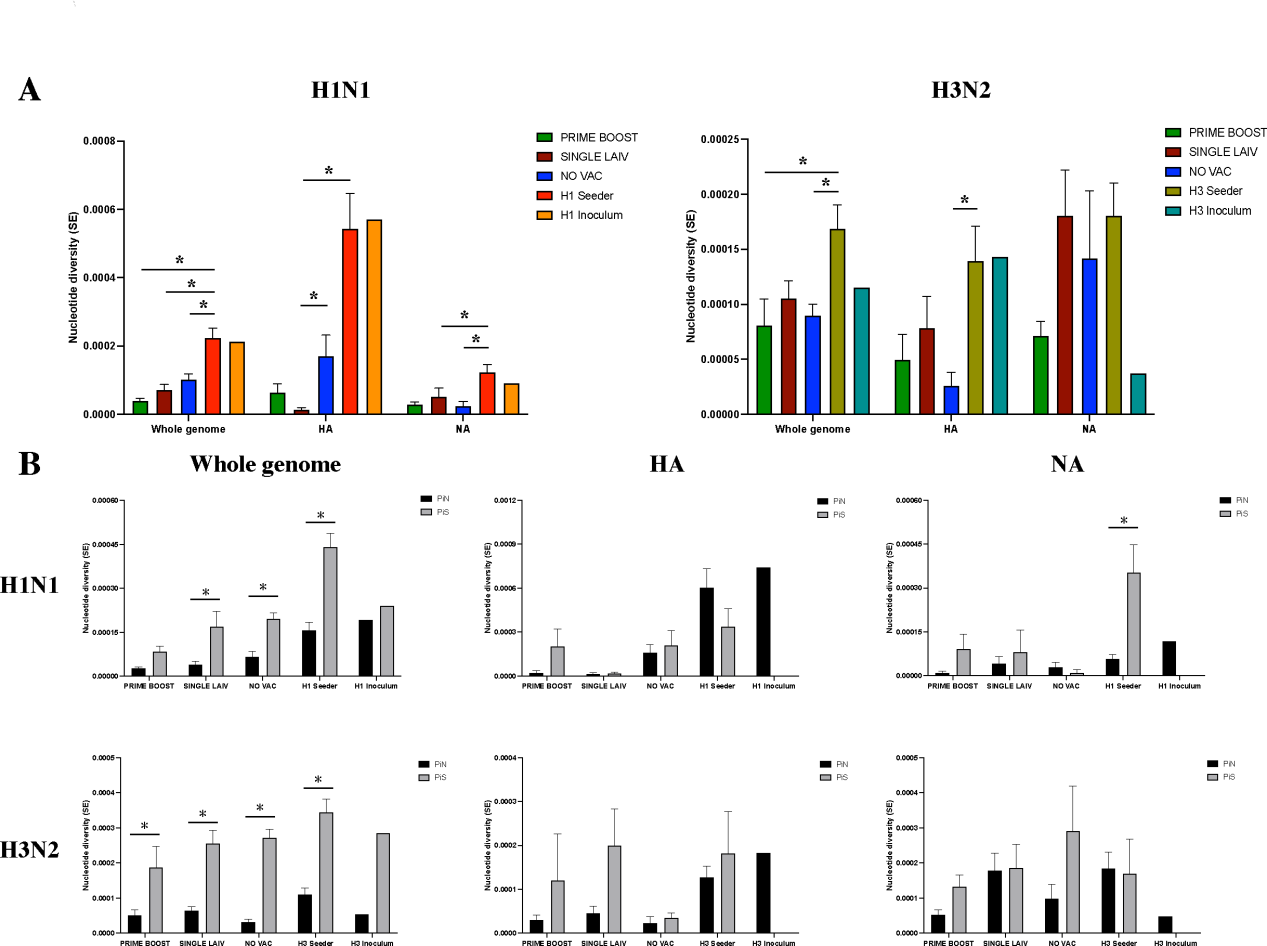
Selective pressure on influenza A virus (IAV) within-host diversity in pigs with different vaccine administrations. (**A**) Nucleotide diversity (Pi) values of the antigenic proteins and the complete genome for H1N1 and H3N2 viruses by treatment groups. We computed the pairwise nucleotide difference per site (Pi) at the IAV whole genome, HA, and NA protein for each sample. The bar plot displays the average Pi value with SE for all the samples combined for each group. The nucleotide diversity between groups was compared by the Kruskal-Wallis rank-sum test and followed by Dunn’s test with the Benjamini-Hochberg correction for multiple pairwise comparisons. The significant results displayed as asterisks. (**B**) The nonsynonymous nucleotide diversity (PiN) and synonymous nucleotide diversity (PiS) at the whole-genome level and at the individual antigenic protein were calculated for each sample. For each virus and treatment group, we computed the mean values of PiN and PiS for all the samples combined (bar plot). The bar plot represents the mean and SE (error bar) for PiN and PiS values of samples collected from each group for the H1N1 and H3N2 viruses. SE was computed by resampling the corresponding values 10,000 times with replacement. The paired t-test was utilized to compare the PiN and PiS for each group. The asterisks show significant results, which prove the alternative hypothesis that PiN ≠ PiS. The Pi, PiN, and PiS values of the antigenic proteins and at the scale of the whole IAV genome for the IAV challenge inoculums are presented for reference but not included in the statistical analysis.

To evaluate whether different vaccine regimens affected the within-host diversity of IAV variants in pigs, we computed the mean number of nucleotide differences per site through all pairwise comparisons of covered gene reads across the target genome to measure the degree of nucleotide polymorphisms of the IAV populations, which is known as nucleotide diversity or Pi (33). We found that both H1N1 and H3N2 viruses exhibited a similar level of nucleotide diversity at the whole-genome and antigenic proteins among contact pigs, except for the higher average Pi value observed on the H1 coding region of NO VAC when compared to SINGLE LAIV pigs (Fig. 3A). However, the two viruses in the corresponding seeder pigs (H1N1 in H1 seeders and H3N2 in H3 seeders) generally exhibited higher nucleotide diversity than the contact pigs, especially at the whole-genome level (Fig. 3A). For the coding regions on the IAV internal genes across all samples together, we did not observe a significant difference in mean Pi values among contact pigs for both viruses, except for the average Pi values of M2 and NS2 in H1N1, and PA-X and NS1 in H3N2 which differed significantly between SINGLE LAIV and NO VAC pigs. For the viruses shed from the seeder pigs, we detected lower Pi values of H1N1 M2 in the H1 seeder compared to NO VAC pigs, and higher Pi values of H1N1 PB1 and M1 in H1 seeder pigs compared to contact pigs from all the three groups. In addition, we found higher Pi values of H3N2 PA-X and NS1 in H3 seeders than in NO VAC pigs, and higher nucleotide diversity of H3N2 PB2 in H3 seeders compared to PRIME BOOST and SINGLE LAIV pigs (Table 2). The Pi values were also calculated for the H1N1 and H3N2 inocula for reference. Except for the IAV coding regions that have no identified SNVs, the Pi values for H1N1 and H3N2 inocula were generally close to the corresponding virus shed from H1 and H3 seeder pigs, respectively (Fig. 3A; Table 2).

**TABLE 2:** Mean values of nucleotide diversity (Pi), nonsynonymous nucleotide diversity (PiN), and synonymous nucleotide diversity (PiS) with standard error in parenthesis, of influenza proteins translated from internal genes for the H1N1 and H3N2 viruses

Protein	Treatment	H1N1					Treatment	H3N2				
		Pi^*a*^	*P*-value^*b*^ (Kruskal-Wallis)	PiN^*a*^	PiS^*a*^	*P*-value^*c*^ (paired t-test)		Pi	*P*-value (Kruskal-Wallis)	PiN	PiS	*P*-value (paired t-test)
PB2	PRIME BOOST	0.000035 (0.000011)	0.393	0.0000077 (0.0000067)	0.00013 (0.000051)	0.148	PRIME BOOST	**0.00012 ^A^** (**0.000078**)	**0.005**	0.000044 (0.000037)	0.00036 (0.00021)	0.146
	SINGLE LAIV	0.00011 (0.000029)		**0.000045** (**0.000024**)	**0.00032** (**0.00010**)	**0.046**	SINGLE LAIV	**0.00014 ^A^** (**0.000027**)		**0.000048** (**0.000015**)	**0.00044** (**0.000097**)	**< 0.001**
	NO VAC	0.000078 (0.000017)		0.000070 (0.000024)	0.00011 (0.000049)	0.597	NO VAC	**0.00022 ^AB^** (**0.000023**)		**0.000026** (**0.000012**)	**0.00086** (**0.000098**)	**< 0.001**
	H1 seeder	0.00014 (0.000042)		0.000087 (0.000023)	0.00031 (0.00013)	0.092	H3 seeder	**0.00032 ^B^** (**0.000036**)		**0.00011** (**0.000029**)	**0.0010** (**0.00013**)	**0.001**
	H1 inoculum **^*d*^**	0.00018		0.00022	0.000064		H3 inoculum **^*d*^**	0.00018		0.000037	0.00066	
PB1	PRIME BOOST	**0.000063 ^A^** (**0.000019**)	**0.001**	0.000050 (0.000015)	0.00011 (0.000051)	0.368	PRIME BOOST	0.000098 (0.000022）	0.073	0.000077 (0.000020)	0.00017 (0.000070)	0.257
	SINGLE LAIV	**0.00011 ^A^** (**0.000028**)		0.000033 (0.0000089)	0.00037 (0.00013)	0.057	SINGLE LAIV	0.000087 (0.000020)		0.000080 (0.000022)	0.00011 (0.000032)	0.381
	NO VAC	**0.00010 ^A^** (**0.000024**)		**0.000043** (**0.000019**)	**0.00030** (**0.000061**)	**0.002**	NO VAC	0.000035 (0.0000075)		0.000028 (0.0000065)	0.000060 (0.000022)	0.171
	H1 seeder	**0.00032 ^B^** (**0.000046**)		**0.00012** (**0.000035**)	**0.0010** (**0.00010**)	**< 0.001**	H3 seeder	0.000097 (0.000024)		**0.000061** (**0.000018**)	**0.00023** (**0.000054**)	**0.018**
	H1 inoculum	0.00025		0.000031	0.0010		H3 inoculum	NA		NA	NA	
PB1-F2	PRIME BOOST	0.000058 (0.000031)	0.216	0.000075 (0.000040)	0.00 (0.00)	0.200	PRIME BOOST	0.000057 (0.000031)	0.549	0.000043 (0.000040)	0.00011 (0.000070)	0.480
	SINGLE LAIV	0.000032 (0.000020)		0.0 (0.0)	0.00015 (0.000093)	0.171	SINGLE LAIV	0.00023 (0.00012)		0.000034 (0.000019)	0.00093 (0.00054)	0.131
	NO VAC	0.0 (0.0)		0.0 (0.0)	0.0 (0.0)	NA	NO VAC	0.000032 (0.000016)		0.000016 (0.000011)	0.000089 (0.000067)	0.322
	H1 seeder	0.00022 (0.00013)		0.00022 (0.00016)	0.00024 (0.00023)	0.946	H3 seeder	0.000040 (0.000023)		0.000026 (0.000024)	0.000094 (0.000086)	0.535
	H1 inoculum	0.0		0.0	0.0		H3 inoculum	NA		NA	NA	
PA	PRIME BOOST	0.000048 (0.0000065)	0.254	0.000036 (0.0000087)	0.000092 (0.000028)	0.240	PRIME BOOST	0.000082 (0.000030)	0.096	0.000045 (0.000015)	0.00022 (0.000086)	0.062
	SINGLE LAIV	0.00012 (0.000050)		0.000086 (0.000038)	0.00026 (0.00015)	0.277	SINGLE LAIV	0.000077 (0.000014)		**0.000048** (**0.000013**)	**0.00018** (**0.000053**)	**0.033**
	NO VAC	0.00010 (0.000030)		**0.000048** (**0.000015**)	**0.00031** (**0.000095**)	**0.019**	NO VAC	0.000073 (0.000019)		**0.000016** (**0.0000076**)	**0.00028** (**0.000074**)	**0.002**
	H1 seeder	0.00018 (0.000036)		**0.000049** (**0.000017**)	**0.00065** (**0.00017**)	**0.008**	H3 seeder	0.00018 (0.000041)		0.00015 (0.000042)	0.00032 (0.00011)	0.220
	H1 inoculum	0.000060		0.0	0.00028		H3 inoculum	0.00011		0.000082	0.00019	
PA-X	PRIME BOOST	0.000033 (0.0000098)	0.067	0.000043 (0.000013)	0.00 (0.00)	0.061	PRIME BOOST	**0.00011 ^AB^** (**0.000056**)	**0.012**	0.00011 (0.000069)	0.00011 (0.000065)	0.945
	SINGLE LAIV	0.000067 (0.000043)		0.000021 (0.000013)	0.00024 (0.00020)	0.355	SINGLE LAIV	**0.00012 ^A^** (**0.000029**)		0.000099 (0.000027)	0.00018 (0.00012)	0.562
	NO VAC	0.000046 (0.000011)		0.000026 (0.000010)	0.00012 (0.000042)	0.075	NO VAC	**0.000033 ^B^** (**0.000016**)		0.000029 (0.000020)	0.000046 (0.000023)	0.601
	H1 seeder	0.00019 (0.000051)		0.000082 (0.000044)	0.00060 (0.00023)	0.074	H3 seeder	**0.00012 ^A^** (**0.000028**)		0.00013 (0.000043)	0.000081 (0.000073)	0.681
	H1 inoculum	0.00010		0.0	0.00047		H3 inoculum	0.0		0.0	0.0	
NP	PRIME BOOST	0.000027 (0.0000081)	0.051	0.000035 (0.000011)	0.00 (0.00)	0.067	PRIME BOOST	0.000050 (0.000015)	0.459	0.000015 (0.0000048)	0.00017 (0.000063)	0.065
	SINGLE LAIV	0.000019 (0.000011)		0.000022 (0.000013)	0.000011 (0.000010)	0.414	SINGLE LAIV	0.00013 (0.000031)		**0.000026** (**0.000011**)	**0.00046** (**0.00012**)	**0.003**
	NO VAC	0.00012 (0.000029)		0.000092 (0.000038)	0.00019 (0.000092)	0.399	NO VAC	0.000098 (0.000030)		**0.000021** (**0.000010**)	**0.00036** (**0.00013**)	**0.031**
	H1 seeder	0.000066 (0.000014)		**0.000039** (**0.000014**)	**0.00016** (**0.000044**)	**0.042**	H3 seeder	0.00013 (0.000043)		**0.000052** (**0.000031**)	**0.00038** (**0.00012**)	**0.040**
	H1 inoculum	0.000061		0.000057	0.000075		H3 inoculum	0.00022		0.0	0.00094	
M1	PRIME BOOST	**0.0000075 ^B^** (**0.0000061**)	**0.008**	0.0000098 (0.0000082)	0.00 (0.00)	0.378	PRIME BOOST	0.000057 (0.000042)	0.218	0.000070 (0.000055)	0.000015 (0.000014)	0.401
	SINGLE LAIV	**0.000051 ^B^** (**0.000033**)		0.000063 (0.000043)	0.000014 (0.000013)	0.360	SINGLE LAIV	0.000026 (0.0000079)		0.000019 (0.0000075)	0.000052 (0.000021)	0.150
	NO VAC	**0.000047 ^B^** (**0.000020**)		**0.000023** (**0.000015**)	**0.00013** (**0.000048）**	**0.038**	NO VAC	0.000028 (0.0000098)		0.000034 (0.000012)	0.0000082 (0.0000080)	0.089
	H1 seeder	**0.00037 ^A^** (**0.000078**)		**0.00048** (**0.00010**)	**0.000020** (**0.000019**)	**0.002**	H3 seeder	0.000096 (0.000029)		0.00011 (0.000040)	0.000063 (0.000058)	0.639
	H1 inoculum	0.00058		0.00077	0.0		H3 inoculum	0.0		0.0	0.0	
M2	PRIME BOOST	**0.00 ^AB^** (**0.00**)	**0.016**	0.00 (0.00)	0.00 (0.00)	NA	PRIME BOOST	0.000044 (0.000032)	0.912	0.00 (0.00)	0.00 (0.00)	NA
	SINGLE LAIV	**0.00 ^A^** (**0.00**)		0.00 (0.00)	0.00 (0.00)	NA	SINGLE LAIV	0.000027 (0.000012)		0.0000065 (0.0000063)	0.00011 (0.000060)	0.092
	NO VAC	**0.00016 ^B^** (**0.000063**)		0.00019 (0.000081)	0.000060 (0.000058)	0.306	NO VAC	0.000019 (0.0000076)		0.00 (0.00)	0.000024 (0.000023)	0.331
	H1 seeder	**0.000021 ^A^** (**0.000020**)		0.000012 (0.000012)	0.000051 (0.000049)	0.341	H3 seeder	0.000014 (0.000013)		0.000018 (0.000016)	0.00 (0.00)	0.363
	H1 inoculum	0.0		0.0	0.0		H3 inoculum	0.0		0.0	0.0	
NS1	PRIME BOOST	0.0000095 (0.0000082)	0.766	0.000012 (0.000011)	0.00 (0.00)	0.391	PRIME BOOST	**0.00011 ^AB^** (**0.000022**)	**0.008**	0.000097 (0.000018)	0.00018 (0.000072)	0.349
	SINGLE LAIV	0.000033 (0.000018)		0.000032 (0.000018)	0.000040 (0.000038）	0.837	SINGLE LAIV	**0.00014 ^B^** (**0.000043**)		0.000071 (0.000021)	0.00039 (0.00016)	0.064
	NO VAC	0.000091 (0.000042)		0.00011 (0.000054)	0.000015 (0.000014)	0.088	NO VAC	**0.000044 ^A^** (**0.000023**)		0.000042 (0.000021)	0.000050 (0.000035)	0.696
	H1 seeder	0.000033 (0.000020)		0.000036 (0.000024)	0.000046 (0.000044)	0.857	H3 seeder	**0.00016 ^B^** (**0.000055**)		0.00018 (0.000076)	0.000098 (0.000064)	0.556
	H1 inoculum	0.000073		0.000096	0.0		H3 inoculum	0.0		0.0	0.0	
NS2	PRIME BOOST	**0.000016 ^AB^** (**0.000014**)	**0.024**	0.000021 (0.000018)	0.00 (0.00)	0.391	PRIME BOOST	0.00012 (0.000033)	0.114	0.000054 (0.000030)	0.00038 (0.00015)	0.094
	SINGLE LAIV	**0.0000063 ^A^** (**0.0000059**)		0.0000080 (0.0000075)	0.00 (0.00)	0.347	SINGLE LAIV	0.000052 (0.000019)		0.000040 (0.000017)	0.00010 (0.000061)	0.354
	NO VAC	**0.00025 ^B^** (**0.00010**)		0.000059 (0.000032)	0.00099 (0.00052)	0.133	NO VAC	0.000039 (0.000020)		0.000030 (0.000018)	0.000074 (0.000042)	0.238
	H1 seeder	**0.00011 ^AB^** (**0.000052**)		0.000056 (0.000025)	0.00033 (0.00017)	0.125	H3 seeder	0.000071 (0.000029)		0.000077 (0.000034)	0.000053 (0.000048)	0.715
	H1 inoculum	0.00013		0.00017	0.0		H3 inoculum	0.0		0.0	0.0	

^*a*^ The values of nucleotide diversity (Pi), nonsynonymous nucleotide diversity (PiN), and synonymous nucleotide diversity (PiS) for each IAV protein are shown as mean (standard error). The standard errors were calculated by bootstrapping the corresponding values 10,000 times with replacement and computing the standard deviation of resampled mean values from 10,000 iterations.

^*b*^ The mean Pi values from pigs by treatment groups were compared for each IAV coding region by the Kruskal-Wallis rank-sum test and the pairwise comparisons were conducted by Dunn’s test. The *P*-values were corrected by the Benjamini-Hochberg method. The statistical significance (*P* < 0.05) is displayed in bold font with different superscripts.

^*c*^ The paired t-tests were performed to compare the mean PiN and PiS values for each protein and treatment group. The *P* values under 0.05 were considered statistically significant and are shown in bold.

^*d*^ The values of Pi, PiN, and PiS for each IAV protein from the virus inoculum (H1 inoculum and H3 inoculum) serve as references and are not included in the statistical analysis.

We further characterized the virus nucleotide polymorphisms by computing the nonsynonymous nucleotide diversity (PiN) and synonymous nucleotide diversity (PiS) for each IAV antigenic protein, internal proteins, and at the whole-genome scale (34). Positive selection is suggested when the values of PiN substantially exceed those of PiS (PiN – PiS >0). On the other hand, an excess of PiS to PiN infers purifying selection (PiN – PiS <0). The approximately equal values of PiN to PiS indicate that virus nucleotides are mainly shaped by genetic drift (PiN – PiS ≈ 0). We generally observed similar or excess PiS to PiN values for both viruses in seeder and contact pigs at HA, NA, and whole-genome scales (Fig. 3B). Except for the H1N1 virus in PRIME BOOST pigs, we detected significant PiN < PiS in the seeder pigs and contact pigs of all three groups for both H1N1 and H3N2 viruses at the whole-genome scale when we combined samples from various time-points together, which is indicative of purifying selection (Fig. 3B). We did not detect statistical significance between the PiN and PiS values on both viruses for seeder and contact pigs on HA and NA proteins, except the significant PiN < PiS on N1 for H1 seeders (Fig. 3B). For the IAV coding regions of internal genes across all the samples, we observed similar PiN to PiS values for all the proteins in H1N1 and H3N2 virus from PRIME BOOST pigs. Compared with PRIME BOOST pigs, we detected a significant PiN < PiS on multiple internal coding regions of H1N1 and H3N2 viruses in SINGLE LAIV and NO VAC pigs (Table 2). For viruses in seeder pigs, significant PiN < PiS were detected in H1N1 PB1, PA, and NP in H1 seeders and H3N2 PB2, PB1, and NP in H3 seeders. We did not find evidence of positive selection in any IAV proteins, or all coding regions combined across the whole data set, except we observed a significantly larger value of PiN to PiS in H1N1 M1 in H1 seeders. Taken together, both H1N1 and H3N2 virus populations in seeder and contact pigs from the various treatment groups were predominantly shaped by genetic drift and purifying selection.

### IAV antigenic variants and evolutionary rates in seeder and contact pigs

**Fig 4:**
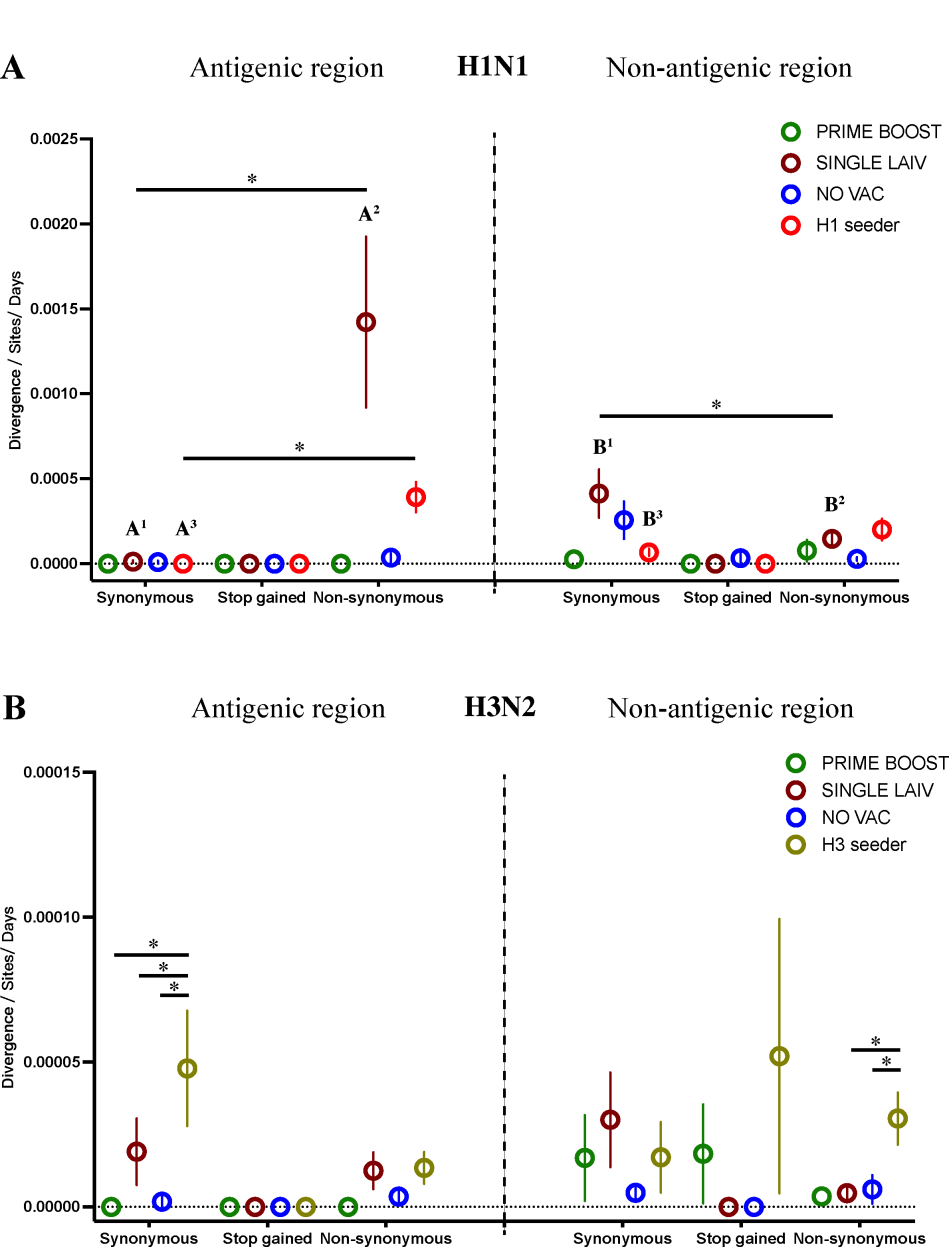
Within-host evolutionary rates on IAV HA antigenic and non-antigenic regions. The within-host evolutionary rates of H1N1 (**A**) and H3N2 (**B**) viruses were calculated for synonymous, stop-gained, and nonsynonymous mutations in HA antigenic and non-antigenic regions by treatment groups. The evolutionary rates for each group and mutation type were displayed as the mean with standard error (SE). The SE was calculated by bootstrapping the corresponding values 10,000 times with replacement and computing the standard deviation of 10,000 bootstrap means. The paired t-test was applied to compare the evolutionary rates between antigenic and non-antigenic regions for each treatment group and mutation type. The statistical significance (*P* < 0.05) of any comparisons is labeled in bold font of different letters with the same superscripts (A^1^ – B^1^, A^2^ - B^2^, or A^3^ – B^3^). The paired t-test was also applied to compare the synonymous and nonsynonymous evolutionary rates for each group in HA antigenic and non-antigenic regions and asterisks displayed significant results (*P* < 0.05). In addition, the Kruskal-Wallis rank-sum test was utilized to compare the evolutionary rates among four different groups for each mutation type and HA region (antigenic or non-antigenic). Dunn’s test performed multiple pairwise comparisons, and the *P*-values were corrected by the Benjamini-Hochberg method. Statistically significant results are shown by asterisks.

Among the samples collected from contact pigs, we did not observe any SNVs located in H1 and H3 antigenic regions in PRIME BOOST pigs. All the detailed information of nonsynonymous SNVs located in H1 and H3 antigenic regions from seeder and contact pigs are summarized in Table S3, based on the identified IAV antigenic regions (35–38). To quantify the speed of variant accumulation on HA proteins, we calculated the evolutionary rates of HA synonymous, stop-gained, and nonsynonymous mutations in antigenic and non-antigenic regions for each sample collected from seeder and contact pigs. For both H1N1 and H3N2 viruses, we did not detect a significant difference in evolutionary rates on any mutation types among contact pigs from the different groups, whether in antigenic or non-antigenic regions. However, we found signs of positive selection in SINGLE LAIV pigs as the H1 nonsynonymous variants in SINGLE LAIV pigs evolved faster in antigenic regions than in the non-antigenic regions. On the contrary, the H1 synonymous mutations accumulated slower in antigenic regions than in non-antigenic regions (Fig. 4A). In addition, significantly higher nonsynonymous to synonymous evolutionary rates within H1 antigenic regions and a significantly larger synonymous to nonsynonymous evolutionary rate within H1 non-antigenic regions were also observed in SINGLE LAIV pigs. For the H1N1 virus in PRIME BOOST and NO VAC pigs and the H3N2 virus in contact pigs of all three groups, we detected similar HA rates between antigenic and non-antigenic regions for all three mutation types.

Similar to SINGLE LAIV pigs, the H1 nonsynonymous mutations from the H1 seeders accumulated faster than synonymous mutations in the antigenic region, and we observed significantly higher H1 synonymous evolutionary rates in non-antigenic regions compared to antigenic regions. However, we did not detect a significant difference in nonsynonymous evolutionary rates between H1 antigenic and non-antigenic regions for H1 seeders. The H3N2 virus in H3 seeders generally exhibited significantly higher evolutionary rates of synonymous mutations in H3 antigenic regions and nonsynonymous mutations in H3 non-antigenic regions than contact pigs from all three groups, with the only exception being the comparison of nonsynonymous rates in H3 non-antigenic regions with PRIME BOOST pigs. However, we did not find differences in evolutionary rates on any type of variants between H3 antigenic and non-antigenic regions in H3 seeders (Fig. 4B).

### Influenza genetic distances and variant transmission between seeder and contact pigs

**Fig 5:**
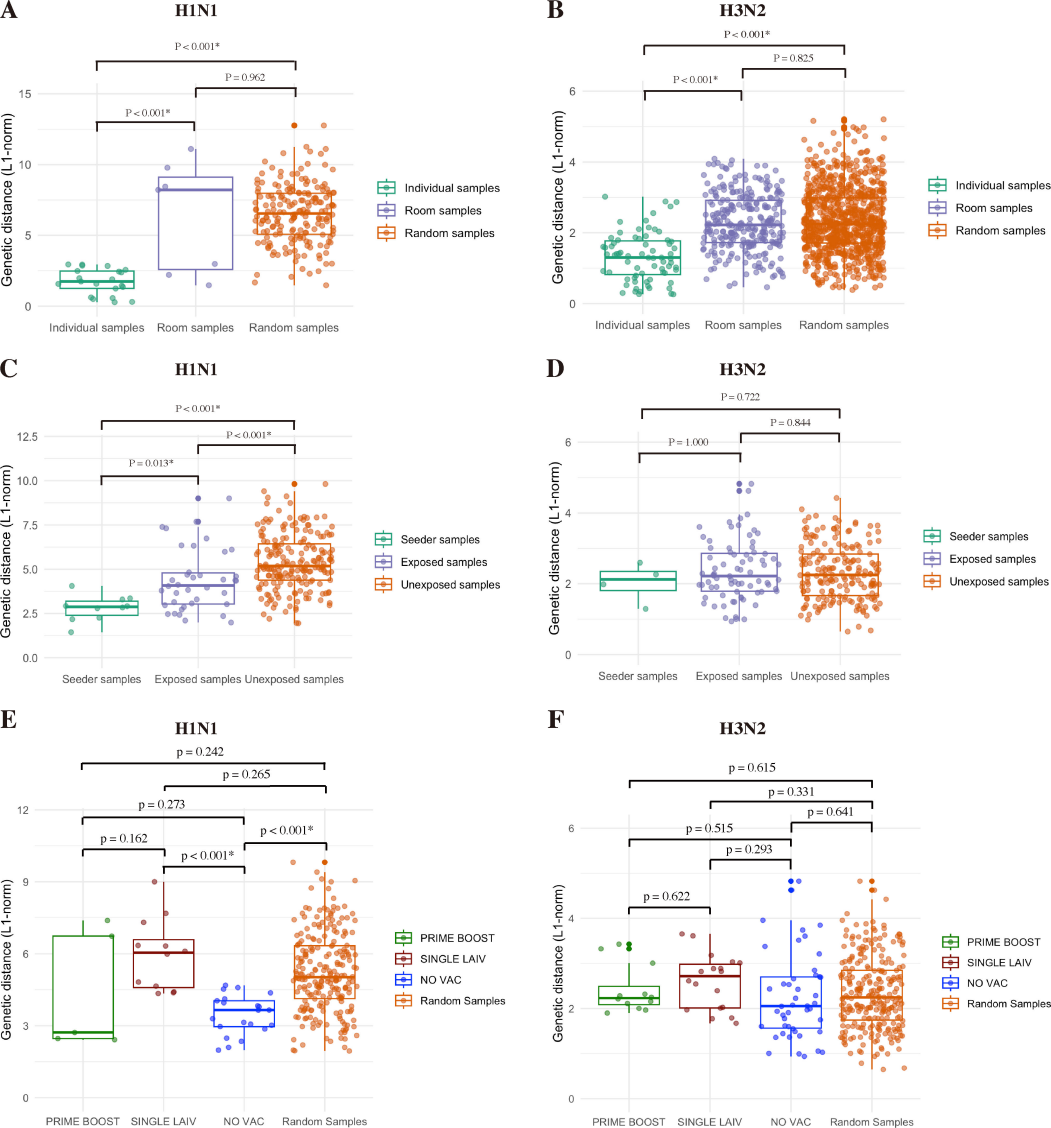
L1-norm pairwise genetic distance of influenza A virus in pigs with various vaccination statuses. Pairwise L1-norm genetic distance between contact pigs within and across the rooms for H1N1 (**A**) and H3N2 (**B**) viruses. The L1-norm values of genetic distance were calculated based on the SNVs across the entire IAV genome for every possible combination of two individual samples (any two samples collected from the same contact pig), room samples (any two samples collected from two contact pigs housed in the same room), and random samples (any possible two sample combinations collected from contact pigs in any rooms). The pairwise L1-norm genetic distance at the whole-genome level of H1N1 (**C**) and H3N2 (**D**) viruses were also computed for every possible two-sample combination collected from the same seeder pig (seeder samples), from one seeder and one contact pig from same room (exposed samples), and from one seeder and one contact pig from different rooms (unexposed samples). The H1N1 (**E**) and H3N2 (**F**) L1-norm pairwise genetic distance in PRIME BOOST, SINGLE LAIV, and NO VAC pigs compared with source viruses shed from seeder pigs. We computed the L1-norm genetic distances across all the virus gene segments for any combinations of two samples, which constitute one sample from either PRIME BOOST (PRIME BOOST), SINGLE LAIV (SINGLE LAIV), or NO VAC (NO VAC) pig and one sample from the seeder pig co-housed in the same room, and any two sample combinations from seeder and contact pigs (Random Samples). Only the samples with complete whole-genome sequences of IAV were enrolled in the analysis, and each dot represents a unique sample pair. An asterisk denoted statistical significance (*P* < 0.05).

**Fig 6:**
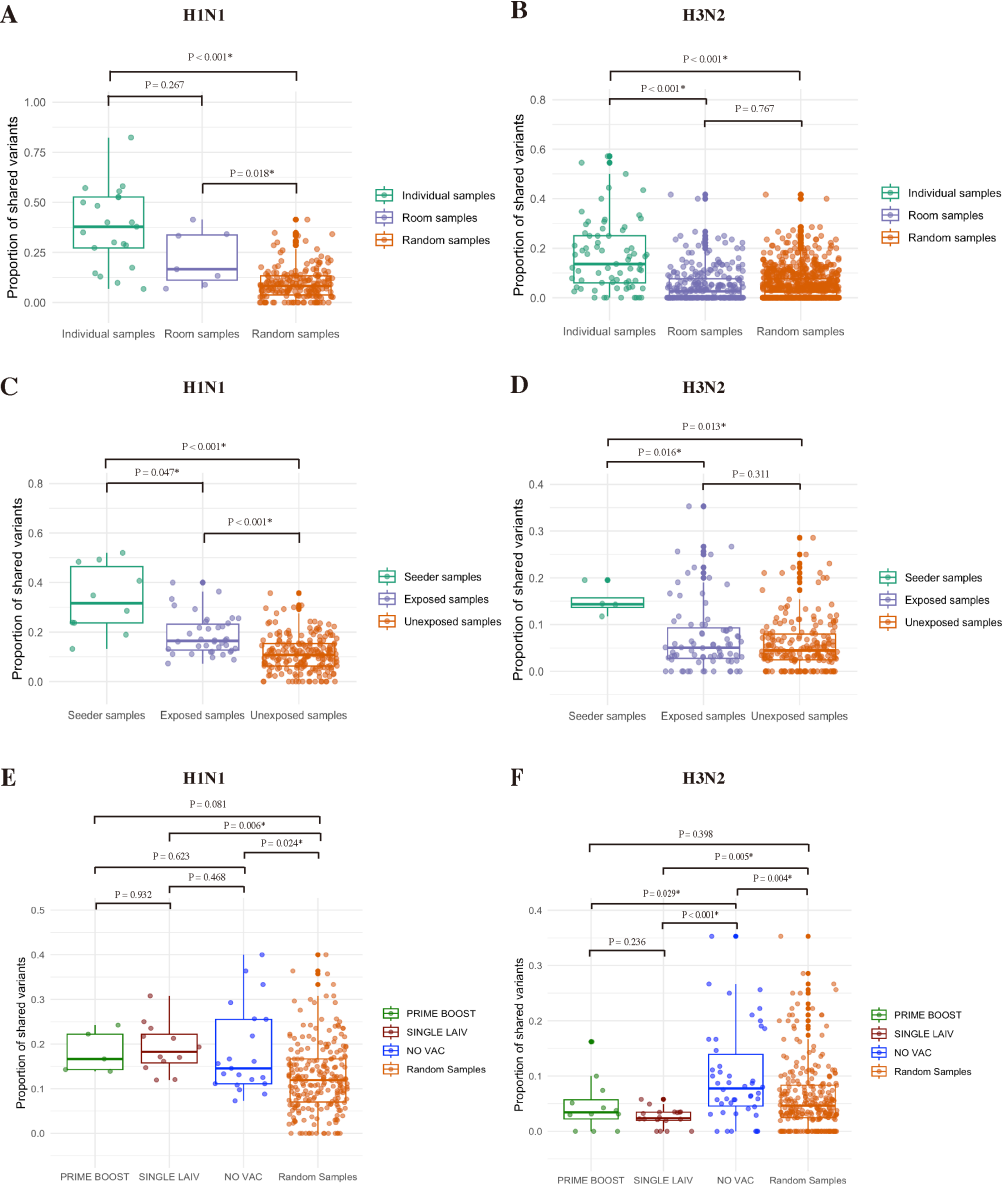
IAV shared variants between pigs housed in the same or different rooms. The proportion of shared variants between contact pigs within and across the room for H1N1 (**A**) and H3N2 (**B**) viruses. The boxplots present the proportion of shared variants, which were calculated based on the SNVs from the whole IAV genome for every possible combination of two individual samples (two samples collected from the same contact pig), room samples (two samples collected from two contact pigs housed in the same room), and random samples (any possible two sample combination collected from two contact pigs in any rooms). The shared variant proportion of any two sample combinations collected from the same seeder pig (seeder samples), from one seeder and one contact pig in the same room (exposed samples), and from one seeder and one contact pig from different rooms (unexposed samples) for H1N1 (**C**) and H3N2 (**D**) viruses. The shared H1N1 (**E**) and H3N2 (**F**) variant proportions in PRIME BOOST, SINGLE LAIV, and NO VAC pigs compared with the source viruses exposed from seeder pigs in the same room. We computed the shared variant proportions at the whole IAV genome scale for any two sample combinations collected from one contact and one seeder pig (Random Samples) and compared with any sample combination made up of one sample from either PRIME BOOST (PRIME BOOST), SINGLE LAIV (SINGLE LAIV), or NO VAC (NO VAC) pigs and one sample from the seeder pig co-housed in the same room. The statistical significance (*P* < 0.05) was denoted by an asterisk.

To assess the extent of IAV genetic variation and what proportion of virus diversity was shared with in-contact pigs from the three treatment groups, we excluded samples that were not fully amplified by whole-genome sequencing. Then, we evaluated the overall degree of IAV genetic variation and shared diversity among the pigs with various vaccination statuses from the same room by computing the genetic distance (L1-norm) and the proportion of shared variants for any two sample combinations collected from the same contact pig (individual samples), from different contact pigs housed in the same room (room samples), and any two samples from two contact pigs housed in any of the rooms (random samples) (Fig. 5A, B, 6A, and B). To know the level of IAV variants that transmit from seeder to contact pigs, we calculated the genetic distance and the proportion of shared variants for any combination of two samples from the same seeder pig (seeder samples), from one seeder and one contact pig housed in the same room (exposed samples), and from one seeder and one contact pig from different rooms (unexposed samples) (Fig. 5C, D, 6C, and D). To further understand how much IAV nucleotide variation and shared diversity was due to the treatment, we compared the IAV SNVs detected in the contact pigs from either PRIME BOOST, SINGLE LAIV, or NO VAC to the background IAV variations of the seeders housed in the same room, and any two sample combinations from seeder and contact pigs (Random Samples) (Fig. 5E, F, 6E, and F).

We observed that both room and random samples exhibited significantly more substantial genetic variation than individual sample combinations in H1N1 and H3N2 viruses (Fig. 5A and B), which infers that there was limited transmission of IAV variants happening between contact pigs co-housed in the same room. Also, we found that unexposed samples had significantly higher genetic distance on the H1N1 virus than exposed samples, and both of them displayed more H1N1 genetic variations than the combinations of seeder samples (Fig. 5C). However, we did not detect any statistical significance on H3N2 genetic variations among seeder, exposed, and unexposed samples (Fig. 5D). These results indicated that the H1N1 variants detected in contact pigs were likely transmitted from seeder pigs, while the de-novo mutations contributed more to the genetic variations of the H3N2 virus in contact pigs. In addition, we found that the H1N1 virus exhibited a significantly more substantial genetic variation in SINGLE LAIV than NO VAC when compared with the IAV background genomic variations from seeder pigs in the same room throughout the study (Fig. 5E). Although the H3N2 virus showed the same trends as the H1N1 genetic distances between the seeder pigs and contact pigs from different treatment groups within the same room, we did not detect significant differences among comparisons (Fig. 5F).

After analyzing the presence and absence of SNVs between individual pigs, we found that both individual and room sample combinations shared significantly higher proportions of H1N1 variants than random sample combinations (Fig. 6A). Although we had a limited sample size of H1N1 room sample combinations, the results suggested that vaccination did not have a significant impact on the transmission of H1N1 variants between the seeders and contact pigs. Unlike the H1N1 virus, we detected a similar proportion of H3N2 variants shared between room sample and random sample combinations and both had significantly lower average proportions compared with individual sample combinations, which suggested that vaccination may have affected the number of different variants shared between contact and seeder pigs (Fig. 6B). In addition, both seeder and exposed sample combinations shared significantly higher proportion of H1N1 variants than the unexposed samples, which implies that the shared H1N1 variants among the contact pigs in the same room were likely derived from the seeder pigs (Fig. 6C). Furthermore, we did not detect significant differences in the proportion of shared H3N2 variants between exposed and unexposed samples, indicating limited impact in the transmission of IAV variants on the overall genetic diversity of the H3N2 virus in contact pigs (Fig. 6D). Moreover, the additional analysis further confirmed the limited impact of vaccination on the transmission of H1N1 variants since we found that the H1N1 virus in the contact pigs of all three groups had similar proportions of variants shared with the seeders from the same room (Fig. 6E). The average proportions of H1N1 variants shared from H1 seeders with SINGLE LAIV and NO VAC pigs were significantly higher than random samples (Fig. 6E). By contrast, the proportions of shared H3N2 variants between contact and seeder pigs varied significantly between treatment groups. We found the average proportion of shared variants of random samples was significantly higher than the SINGLE LAIV pigs but lower than the NO VAC when comparing the shared variants with the seeder pigs from the same room. In addition, we found that the H3N2 virus in both PRIME BOOST and SINGLE LAIV pigs shared a significantly lower proportion of variants than in the NO VAC pigs when compared with the source viruses from seeder pigs housed in the same room (Fig. 6F).

## DISCUSSION

Characterizing the patterns of IAV genetic variation within and between pigs of different vaccination statuses allows for a better understanding of how vaccination affects virus evolution and transmission in populations, which is essential for disease modeling and controlling IAV in pigs. In this study, we performed next-generation sequencing of IAV directly on 77 nasal swabs *a priori*, that is, without first isolating the virus in cell culture. The swabs were collected at multiple time points from seeder pigs shedding H1N1 and H3N2 viruses and from pigs vaccinated with various immunization regimens that were in contact with the seeder pigs after commingling them together. Consistent with the human studies (24, 27), we observed that the dominance of low-frequency H1N1 and H3N2 variants was generally shaped by purifying selection and genetic drift in pigs regardless of vaccination status. However, we found that vaccination can potentially reduce the degree of H3N2 virus diversity shared between seeders and contact pigs. Both H1N1 and H3N2 viruses in SINGLE LAIV vaccinated pigs generally acquired more extensive genome changes than in non-vaccinated pigs during the observation period. In addition, we observed significantly higher nonsynonymous and lower synonymous evolutionary rates in H1 antigenic regions than in the non-antigenic regions in pigs that received a single-dose live vaccine. Overall, we found that the non-sterilizing immune response induced by a heterologous vaccine was unable to limit virus replication, facilitated genetic variation, and exerted selective pressure on H1 antigenic regions.

Compared with wild birds, fewer IAV subtypes are circulating in pigs, mainly consisting of H1 and H3 subtype viruses that belong to different HA groups with divergent phylogenetic clusters (39, 40). As a result, pigs have a high potential of becoming consecutively infected by H1 and H3 IAVs. We found that IAV co-infections with heterologous subtypes arose in about 43% of seeder pigs and 45% of SINGLE LAIV and NO VAC pigs combined, which resulted in complex and extended IAV shedding patterns over time. However, only a few incidents of IAV co-infections were observed in pigs that received two doses of vaccines likely due to the presence of a more robust protective immunity. Overall, our results showed that prime-boost vaccination could decrease the chance of pigs becoming infected by multiple subtypes consecutively and concomitantly. This observation, if supported under field conditions, is especially noteworthy when considering that thousands of pigs of various ages, immune statuses, and infection histories may be housed together in the same environment. Management practices employed in swine farms that include new animal introduction and movement of animals within farms combined with high susceptibility and extended shedding of genetically distinct viruses facilitate the continuous infection of susceptible pigs (41, 42). With continuous exposure to multiple IAV subtypes, the infected pigs have limited time to develop protective immunity and can easily acquire another infection by a distinct subtype, which, in turn, promotes the circulation of diverse IAV subtypes at the population level and further increases the risk of IAV reassortment in pig herds (43).

Host immunity plays a significant role in driving positive selection of influenza virus at the global and individual host scale (44). Even though the single-dose LAIV vaccine elicited a certain amount of HA-specific cell-mediated immunity, we detected minimal antibody response specific to the challenge strains, and the single LAIV-vaccinated pigs showed similar disease severity compared to unvaccinated pigs. The poor immunity and protection induced by the LAIV could result from the antigenic mismatch, especially considering that the HAs of the vaccine and challenge strains belonged to two distantly related phylogenetic clusters (H1 vaccine—gamma2 beta-like or clade 1A.2.3-like vs H1 challenge—clade 1A.3.3.3, H3 vaccine—cluster I vs. H3 challenge—cluster human-like) (45, 46); the poor immunogenicity from the single-dose administration; or the lack of sufficient serum antibody production due to the intranasal vaccine administration. The apparent non-sterilizing immunity observed in pigs vaccinated with the single LAIV vaccine may facilitate both challenge viruses accumulated more mutations across the whole genome compared to unvaccinated pigs during the same observation period. This is similar to the findings of multiple IAV studies where the non-sterilizing immune response induced by vaccination or natural virus infection promoted the emergence and selection of antigenic variants (47–49). Compared with the virus shed from non-vaccinated pigs, the H1N1 viruses detected on single LAIV-vaccinated pigs shared similar proportions of variants but exhibited significantly stronger extent of genome deviations from the source viruses that shed from H1 seeders. Although the extent of genome variations among the H1N1 virus harbored in LAIV-vaccinated pigs is also higher compared to the virus that emerged in the non-vaccinated pigs, the differences were not statistically significant. Therefore, the more extensive genome changes of the H1N1 variants in SINGLE LAIV pigs compared with source viruses were likely due to the presence of a large proportion of high-frequency variants, with certain high-frequency variants fixed among pigs during the infection and repeatedly identified on multiple samples, possibly due to variant selection processes. However, that was not the case for the H3N2 virus. We observed the H3N2 virus sampled from single LAIV-vaccinated pigs showed significantly higher genome variations than the virus from non-vaccinated pigs. However, we did not observe a more significant genetic distance on the H3N2 virus from the single LAIV-vaccinated pigs than NO VAC pigs when compared to the source virus shed from seeder pigs. As a result, the greater extent of genetic variation among the H3N2 virus that emerged in single LAIV-vaccinated pigs may be attributed to the generation of a larger number of diverse low-frequency IAV variants in single LAIV-vaccinated pigs after the transmission of viruses from H3 seeders.

As an effective positive selection requires virus populations to accumulate sufficient quantity and concentration of diverse variants within hosts, the intensity of selective pressure in individual hosts relies highly on the duration of virus infection, mutation rates, and demographic factors like virus population expansion (50–52). This explains our observation that the H1N1 virus in single live-vaccinated pigs exhibited significantly higher nonsynonymous and lower synonymous evolutionary rates in H1 antigenic sites than non-antigenic sites, and their variant frequencies across the whole genome were significantly higher than in unvaccinated pigs that exhibited an increasing trajectory during the most time of study, which are signs of positive selection. However, these observations were not true for the H3N2 virus in single live-vaccinated pigs. Even though we detected a higher total number of H3N2 variants in single live-vaccinated pigs than in unvaccinated pigs, the average variant frequency was low and exhibited a flat trajectory during infection. Previous observations could explain that H1N1 was less transmissible among seeder and contact pigs (32) and had lower growth kinetics on MDCK cells than the H3N2 virus (Fig. S1). When the fitness landscape of the environment changes by drug treatments or vaccine-induced immunity, the less adapted virus may have high mutation rates and faster expansion of the variant populations to accelerate variant accumulation and maximize its chance to replicate in the new environment, which strengthens selection (21, 53, 54). Even though the H1N1 virus in our study had a higher frequency of variants during infection, the positive selection pressure was barely intense enough to be detected at the gene segment or whole-genome level since pigs were infected for only a few days. Thus, compared with the H1N1 virus, the selection pressure on the H3N2 virus was even weaker and much more challenging to detect.

The variants generated within hosts must get through transmission bottlenecks between infected hosts to contribute to IAV evolution at the community or even global level (55). Transmission bottlenecks play an essential role in virus evolutionary dynamics as tight transmission bottlenecks promote de-novo mutations, reduce selection efficiency, and finally slow down the adaptive progression of the virus (55, 56). Therefore, the stringency of transmission bottlenecks determines the composition of mutations in the recipients, affecting viral evolution by continually adjusting the structure of the virus population circulating among susceptible hosts. Multiple studies have estimated the stringency of IAV transmission bottlenecks in humans with varying results, possibly due to different experimental designs, the background of human subjects, the criteria to define transmission pairs, and the sequencing technique (24, 26, 54). Few studies have measured the transmission bottlenecks of IAV in pigs except for one study that revealed the loose transmission bottlenecks present in both naïve and vaccinated pigs based on HA1 sequence data (30). However, in our study, the transmission of IAV variants varied between the IAV subtypes. The transmission of H1N1 variants between seeder and contact pigs was concordant with the observations in a previous publication (30) as we found that vaccination had limited impact on IAV transmission bottlenecks and the H1N1 variant transmission contributed significantly to the virus genomic diversity in the contact pigs. By contrast, we observed that the overall amounts of shared variants of the H3N2 virus between co-housed seeder and contact pigs were insignificant, and the H3N2 variants detected in contact pigs are more likely the result of de-novo mutations rather than the variants being transferred between hosts and expanded in the recipient pigs. In addition, the whole genome data of our study implied that vaccination decreases the H3N2 shared diversity between seeder and contacted pigs, which could constrain the spread and selection of novel influenza variants. In turn, this information suggested that a stringent transmission bottleneck may have been present during the transmission of H3N2 variants, especially between seeder and vaccinated contact pigs in the same room.

Pig populations in US swine farms are highly dynamic due to management practices (41, 42). We hypothesize that pig allocation in our study resembles, in part, pig populations under field conditions given the varying degrees of immunity in the pigs and that the results of this study reflect in part the transmission and selection of IAV variants between and within pigs in swine farms. The virus mutation rate and the longevity of IAV infections in pigs greatly impact the effectiveness of selection processes on virus populations in individual hosts. Given that the US swine industry contains around 74 million pigs and has a high morbidity of IAV infections (57), even low variant transmission possibilities with weak selection processes in individual pigs could drive IAV evolution globally (24). Therefore, unraveling how vaccine-induced or natural immunity affects the transmission of IAV variants and selection is essential for developing more effective influenza control programs to eliminate IAV from pig farms and further benefit human public health. Our study reveals the critical role vaccination plays in IAV evolution and diversity in the overall landscape of IAV ecology in pig populations. Future investigations are needed to validate our results in large-scale animal populations under field conditions.

## MATERIALS AND METHODS

### Specimen collection in pigs from a co-infection model

We characterized the IAV populations in nasal swabs archived from a swine vaccine challenge study where the protective efficacy of multiple prime-boost vaccination protocols against simultaneous H1N1 and H3N2 IAV challenges using a seeder pig co-infection model was evaluated (32). The collection procedures of swine specimens were approved by the University of Minnesota Institutional Animal Care and Use Committee (Protocol ID: 1712-35407A) and the Institutional Biosafety Committee (Protocol ID: 1508-32918H).

Briefly, a total of 84 pigs seronegative to IAV, porcine reproductive and respiratory syndrome virus (PRRSV), and *Mycoplasma hyopneumoniae* were enrolled in the study. Seventy of them were randomly assigned into seven treatment groups and vaccinated following different protocols that included a commercial whole-inactivated vaccine (COM), an autogenous whole-inactivated vaccine (AUT), and a live attenuated vaccine (LAIV). The COM vaccine contained one H1N1 (clade 1A.3.2, 95.1% H1 amino acid identity with challenge virus), one H1N2 (clade 1B.2.2.2, 78.4% H1 amino acid identity with challenge virus), and two H3N2 strains (cluster IV-A, 87.1% H3 amino acid identity; cluster IV-B, 88.2% H3 amino acid identity compared with challenge virus). The AUT vaccine included one H1N1 (clade 1A3.3.3, 96.5% H1 amino acid identity with challenge virus), one H1N2 (clade 1B.2.2.1, 78.6% H1 amino acid identity with challenge virus), and one H3N2 (cluster human-like, 99.1% H3 amino acid identity with challenge virus) viruses. The LAIV vaccine was made up of one H1N1 (gamma2 beta-like or clade 1A.2.3-like, 93.3% H1 amino acid identity with challenge virus) and one H3N2 (cluster I, 89.2% H3 amino acid identity with challenge virus) strain. Fifty pigs from four whole inactivated vaccinated (WIV) groups (COM/COM, AUT/AUT, AUT/COM, and COM/AUT) and one unvaccinated positive control group (NO VAC/CHA) were housed together and evenly distributed in five rooms. Twenty pigs from two LAIV treatment groups (LAIV/NONE and LAIV/COM) were distributed evenly into two rooms. In addition to the contact pigs, 14 naive pigs served as seeder pigs which were inoculated by either an H1N1 (A/swine/Minnesota/PAH-618/2011) or H3N2 (A/swine/Minnesota/080470/2015) virus. All the 7 H1N1 and 7 H3N2 seeders were distributed as a pair in each room and commingled with the contact (treatment) pigs at approximately 48 hours post-inoculation (0 days post contact, dpc), when they were confirmed to be infected and shed the primary inoculated virus by IAV matrix rRT-PCR. The seeder pigs became the source of IAV transmission to the contact pigs. Nasal swabs were taken daily from all pigs from 2 to 6 dpc.

Immunological parameters were also assessed. Briefly, we barely identified detectable HA-specific antibodies measured by hemagglutination inhibition (HI) against H1N1 and H3N2 challenge viruses in non-vaccinated (NO VAC/CHA pigs) pigs and pigs that received a single dose of the live vaccine (LAIV/NONE pigs). By contrast, pigs that received two doses of whole-inactivated vaccines (WIV; including COM/COM, AUT/AUT, AUT/COM, and COM/AUT pigs) and the combination of one live and one whole-inactivated vaccine (LAIV/COM pigs) had significantly higher average HI titers against both challenge viruses than NO VAC/CHA pigs and LAIV/NONE pigs (32). The pigs in the various treatment groups exhibited a similar level of T-cell-mediated immune response, as the pigs had a close number of H1- and H3-specific IFN-γ-secreting cell counts measured by ELISPOT, except for the higher number of H1-specific IFN-γ cells counts observed in NO VAC/CHA pigs compared to COM/COM and AUT/AUT pigs, which was likely due to the severe virus infections. As a result, we observed similar protection among pigs that received different two-dose vaccine combinations. Each combination of these double-dose vaccine treatments generally exhibited superior protection compared to LAIV/NONE and NO VAC/CHA pigs, based on the amount of virus shed from lungs and nasal cavities, whether they were measured by IAV matrix gene real-time PCR or virus quantification (TCID_50_) (32).

In this study, we only focused on measuring the transmission and diversity of IAV variants collected from the nasal cavities of contact pigs since they represent better the virus population being shed during transmission. For analytical purposes, we categorized the contact pigs into three treatment groups named (a) PRIME BOOST (COM/COM, AUT/AUT, COM/AUT, AUT/COM, and LAIV/COM pigs), (b) SINGLE LAIV (LAIV/NONE pigs), and (c) NO VAC (NO VAC/CHA pigs) groups. In addition, we sequenced the nasal swabs collected from the H1N1 and H3N2 challenged seeder pigs at and after 2 dpc to trace the dynamics of genetic diversity for the inoculated viruses in the seeder pigs and compared them with the IAV variants from the contact pigs (only H1N1 gene reads sequenced from H1 seeders’ nasal swabs and the H3N2 gene reads sequenced from H3 seeders’ nasal swabs were used in this study). The challenge inoculum was also sequenced and the detailed information of the vaccines used in the study can be found in (32).

### Influenza virus detection in samples

All the nasal swabs were suspended in IAV transport media and frozen at −80℃ within the same day after collection. Viral RNA was extracted from the nasal swabs using the MagMax Viral RNA isolation kit (Ambion, Life Technologies, USA). We performed the one-step rRT-PCR that targets the IAV M gene on the extracted RNA using the 7500 Fast real-time PCR system (Life Technologies), and samples with Ct values below 35 were included for next-generation sequencing. IAV multiplex rRT-PCR targeting the H1 and H3 genes was conducted using the VetMAX—Gold SIV Subtyping Kit to identify the co-infection patterns by both viruses in pigs (Life Technologies, Austin, TX, USA). Based on the manufacturer’s instructions, the samples were considered as positive and suspect if their Ct values were under 38 and between 38 and 40, respectively. In this study, we set the Ct value of 39 as the threshold for identifying the positive samples.

### Complementary DNA amplification, PCR, and next-generation sequencing

We conducted the one-step reverse transcription-PCR amplification on extracted viral RNA from the selected nasal swabs (Ct value of IAV matrix gene rRT-PCR below 35) to synthesize viral complementary DNA (cDNA) of each sample, using the SuperScript III One-Step RT-PCR system with High Fidelity Platinum Taq DNA Polymerase (Invitrogen, Life Technologies, USA) and IAV universal primers (10 μM MBTuni-12M and MBTuni-13) based on methods published before (58). The quality of the amplified IAV cDNA genome from each sample was visually inspected by gel electrophoresis and the eligible PCR products were cleaned up by Qiagen QIAquick PCR Purification Kit (QIAGEN, USA). Purified PCR products were sent to the University of Minnesota Genomics Center (UMGC) for next-generation sequencing. Briefly, submitted PCR products were first checked by Nanodrop and PicoGreen DNA Quantification (ThermoFisher Scientific, Waltham, MA) (59, 60). Sequencing libraries were created by UMGC staff using the Nextera DNA XT Sample Preparation Kit (Illumina, San Diego, CA, USA). The concentration of each barcoded library was quantified by the Quant-iT PicoGreen dsDNA Assay Kit (Invitrogen), multiplexed, and sequenced by the ILLUMINA NextSeq or Miseq platforms on 150 bp pair-end mode (ILLUMINA, San Diego, CA, USA) after pooling the barcoded libraries together at equal nanomolar concentration.

### Raw reads processing, mapping, and variant identification

We analyzed the raw gene reads released from UMGC using available bioinformatic tools at the University of Minnesota Supercomputing Institute (MSI). The bioinformatic pipelines used for IAV variant detection in nasal swabs were adapted from the variant calling strategy described elsewhere (25). After quality check by Fast-QC, we used Trimmomatic to trim the raw reads by scanning the gene reads in five base pairs’ sliding window and removing the barcodes, adaptors, and the bases with Q-score below three from both terminals of the reads until the average Q score of bases in the sliding window was above 30 (61, 62). We discarded the trimmed reads with lengths below 75 bp from further analysis. Trimmed reads were mapped against the reference genomes, that is, the H1N1 and H3N2 IAV challenge viruses, using the Geneious Reads Mapper (63). More specifically, the reference genomes used were sequenced from nasal swabs collected from H1 and H3 seeder pigs at 2 days post-challenge, that is, 0 days post-contact (0 dpc), which was before contact with the other pigs. The IAV whole-genome sequences of the H1N1 and H3N2 challenge strains that are used for variant identification have been deposited in GenBank with accession numbers MT 377710 to MT377725. We exported the mapped reads in SAM format, sorted them by Picard Sortsam, and removed identical reads by Picard MarkDuplicates (http://broadinstitute.github.io/picard/) (64). The pileup files were prepared by samtools mpileup (http://www.htslib.org/doc/samtools-mpileup.html) and used for variant calling by VarScan (https://github.com/dkoboldt/varscan) (65). The inclusion criteria of IAV variants used for our analysis were similar to those previously described (25) in that we also required a minimum sequencing depth of 100 reads, a minimum average variant base quality score of 30, a variant frequency >1%, and variants needed to be detected in both forward and reverse reads. Due to the close genetic distance of the internal genes between the two challenge viruses, variant calling reports from samples containing both challenge viruses were further checked to remove false-positive variants that result from gene reads mis-mapping to template references (H1N1 gene reads mapped to the H3N2 template and vice versa). Finally, we checked the originally mapped sequence reads manually and removed the false positive SNVs accordingly if the identified polymorphic nucleotides were due to random mapping errors. The final IAV variants were annotated based on their mutation types (nonsynonymous, stop-gained, or synonymous). In addition to the SNVs detected in the samples from contact pigs, we also included the identified SNVs from the seeder pigs in the analysis to infer the background genomic variations of the source viruses in the study. Both SNVs identified in contact and seeder pigs were called using the same bioinformatic pipeline and criteria.

### Bioinformatics analysis

All the bioinformatics and evolutionary analyses were performed on the final reports of IAV variants identified based on the described inclusion criteria. We calculated the IAV mutation rate for each sample as the total quantity of identified polymorphic nucleotide sites divided by the total number of nucleotide sites of all sequenced IAV gene segments in a sample. The L1-norm genetic distance and shared proportion of variants for H1N1 and H3N2 virus was calculated at the whole-genome level, which includes the nucleotide mutations identified at the coding regions of PB2, PB1, PB1-F2, PA, PA-X, HA, NP, NA, M1, M2, NS1, and NS2. If a single mutation presented in both coding regions, the mutation was counted twice. The mean number of pairwise differences per nucleotide site or nucleotide diversity (Pi) and their nonsynonymous and synonymous partitions (PiN and PiS) were computed for each sample across IAV individual coding regions and all coding regions combined together by SNPGenie (https://github.com/chasewnelson/SNPGenie) (66). Virus genetic distances between any sample pairs were displayed in the L1-norm (Manhattan Distance) by computing the sum of absolute values of frequency differences on identified SNVs across all the polymorphic nucleotide sites from two samples during the pairwise comparisons (26). The proportion of shared variants of any two samples was computed as the number of shared variants multiplied by two and divided by the total number of variants identified in these two samples (67).

The within-host evolutionary rates were calculated for the synonymous, stop-gained, and nonsynonymous SNVs located in HA antigenic and non-antigenic regions for each sample using the methods from a published study (68). The HA evolutionary rates were computed by summing the frequencies of SNVs by mutation types and HA regions (antigenic or non-antigenic regions), then divided by available sites and number of days post-contact with the seeder pigs when the samples were collected. The HA coding regions of H1N1 and H3N2 challenge viruses used in this study are both comprised of 1,701 nucleotide sites, and there are 53 (159 nucleotide sites) and 131 (393 nucleotide sites) amino acid positions described as antigenic regions for H1 and H3 subtype influenza viruses, respectively (35–38). The available nucleotide sites for synonymous, stop-gained, and nonsynonymous mutations were computed by multiplying the total nucleotide sites of HA antigenic or non-antigenic regions by 25, 3, and 72%, respectively (68, 69).

### Statistical analysis

We performed the statistical analysis using R program version 3.6.2 (70). We computed the standard error (SE) of Pi, PiN, and PiS values for each treatment group and SE of within-host evolutionary rates for each treatment group and mutation type, by bootstrapping the corresponding values 10,000 times with replacement and computed the mean number of resampled values during each iteration. The SE is shown as the standard deviation of the 10,000 resampled means.

The one-way ANOVA test was performed to compare the mean values of variant frequency between treatment groups at 4, 5, and 6 dpc for both H1N1 and H3N2 viruses, the Tukey’s HSD was conducted to make the pairwise comparisons. Since no samples collected from PRIME BOOST pigs at 2 and 3 dpc were successfully sequenced for either H1N1 or H3N2 viruses, the student t-test was applied instead to compare the average values of variant frequency between SINGLE LAIV and NO VAC pigs. The Kruskal-Wallis rank-sum test followed by Dunn’s test with Benjamini-Hochberg correction was applied to compare the mean nucleotide diversity (Pi) values among seeder and treatment groups at individual IAV coding regions or whole-genome level. The paired t-tests were performed for each treatment group to compare the significant differences between the average values of PiN and PiS at each coding region or all the coding regions combined. For the within-host evolutionary rates, the paired t-test was applied to compare the significant differences in the mean HA rates between the HA antigenic and non-antigenic regions for each group and mutation type. For each treatment group, the paired t-test was used to test whether there is a significant difference between the average synonymous and nonsynonymous evolutionary rates in HA antigenic and non-antigenic regions. The average within-host evolutionary rates by mutation types and HA regions were compared between groups using Kruskal-Wallis rank-sum test, and followed by Dunn’s test with Benjamini-Hochberg corrections.

The comparisons of L1-norm genetic distance and shared proportion of variants for H1N1 and H3N2 virus between different groups were tested by the Kruskal-Wallis rank-sum test, and followed by Dunn’s test for multiple group comparisons. The *P*-values were corrected by the Benjamini-Hochberg method and a *P*-value < 0.05 was considered statistically significant.

## Data Availability

All the raw sequence reads of influenza viruses generated in this study have been deposited in the Sequence Read Archive (SRA) of the National Center for Biotechnology Information (NCBI) under BioProject accession numbers PRJNA935082 and PRJNA824122.

## SUPPLEMENTAL MATERIAL

Supplemental material jvi.01245-24-s0001.docx Fig. S1 and Tables S1 to S3.

ASM does not own the copyrights to Supplemental Material that may be linked to, or accessed through, an article. The authors have granted ASM a non-exclusive, world-wide license to publish the Supplemental Material files. Please contact the corresponding author directly for reuse.
