# Supplementary material for: Quantifying the impact of vaccination on transmission and diversity of influenza A variants in pigs
Source: J Virol. 2024 Nov 12;98(12):e01245-24. doi: 10.1128/jvi.01245-24 (PMC11651001; doi:10.1128/jvi.01245-24)
Supplement: Supplemental material — Fig. S1 and Tables S1 to S3. [file jvi.01245-24-s0001.docx]

**Fig S1. Growth kinetics of H1N1 and H3N2 challenge viruses in Madin Darby canine kidney (MDCK) cells.** MDCK cells were infected with H1N1 (A/swine/Minnesota/PAH-618/2011) and H3N2 (A/swine/Minnesota/080470/2015) viruses at the MOI of 0.001 TCID_50_/cell. The growth curves were determined by the virus titers of the supernatants from MDCK cells at 12, 24, 36, 48, 60, and 72 hours post-infection. The virus titers are shown as means with standard deviations (three independent replicates). The dashed line indicates the detection limit of virus quantification measured by TCID_50_/ml.

**Table S1. Comparison of vaccine-induced immunity against challenge viruses in pigs from different groups.**

| Immunity | Group | Challenge virus | | Measurement |
| --- | --- | --- | --- | --- |
|  |  | H1N1  Mean (SEM) ^a^ | H3N2  Mean (SEM) ^a^ |  |
| Humoral immunity | PRIME BOOST | 47.9 ^A^  (6.6) | 210.0 ^A^  (28.5) | Hemagglutination Inhibit Assay  (HI titer) |
|  | SINGLE LAIV | 2.0 ^B^  (0.0) | 2.0 ^B^  (0.0) |  |
|  | NO VAC | 2.0 ^B^  (0.0) | 6.2 ^B^  (1.9) |  |
| Cell meditated immunity | PRIME BOOST | 93.3 ^A^  (13.8) | 81.0 ^A^  (15.6) | ELISPOT  (IFN-γ secreting cell count) |
|  | SINGLE LAIV | 86.7 ^A^  (33.5) | 72.2 ^A^  (28.5) |  |
|  | NO VAC | 318.8 ^B^  (84.4) | 185.0 ^A^  (71.6) |  |

^a^ The HI titers and ELISPOT IFN-γ secreting cell counts against H1N1 and H3N2 viruses from pigs in different treatment groups were compared by Kruskal-Wallis rank-sum test and followed by Dunn’s test with Benjamini-Hochberg corrections. The samples that have HI titers below the detection limit were assigned a titer of 2. The statistical significance (p < 0.05) is shown by different superscripts (A, B).

**Table S2. Comparison of cycle threshold values of influenza A virus in nasal swabs measured by influenza hemagglutinin subtyping real-time PCR from pigs in different groups.**

| Virus | Group | Days Post Contact  Mean (SD) ^a^ | | | | | |
| --- | --- | --- | --- | --- | --- | --- | --- |
|  |  | Nasal swab | | | | | BALF |
|  |  | 2 dpc | 3 dpc | 4 dpc | 5 dpc | 6 dpc | 7 dpc |
| H1N1 | PRIME BOOST | 39.9 ^A^  (0.7) | 39.9 ^A^  (0.6) | 39.8 ^A^  (0.8) | 39.8 ^A^  (1.3) | 39.7 ^A^  (1.6) | 39.8 ^A^  (0.7) |
|  | SINGLE LAIV | 39.6 ^A^  (1.3) | 38.7 ^A^  (2.9) | 38.2 ^A^  (3.9) | 36.3 ^B^  (5.0) | 38.2 ^AB^  (3.7) | 34.4 ^B^  (5.3) |
|  | NO VAC | 39.0 ^A^  (2.0) | 38.5 ^A^  (3.1) | 37.1 ^A^  (4.7) | 35.7 ^B^  (6.4) | 37.1 ^B^  (3.8) | 32.4 ^B^  (6.0) |
| H3N2 | PRIME BOOST | 40.0 ^A^  (0.0) | 39.8 ^A^  (1.1) | 39.3 ^A^  (2.0) | 39.2 ^A^  (2.2) | 39.2 ^A^  (2.4) | 38.2 ^A^  (3.9) |
|  | SINGLE LAIV | 37.2 ^B^  (3.7) | 36.2 ^B^  (5.0) | 35.3 ^A^  (6.2) | 35.7 ^A^  (5.7) | 37.2 ^AB^  (3.6) | 33.6 ^A^  (6.5) |
|  | NO VAC | 37.9 ^B^  (3.5) | 37.0 ^B^  (5.0) | 35.7 ^A^  (5.6) | 34.2 ^A^  (7.7) | 34.5 ^B^  (7.1) | 33.8 ^A^  (8.0) |

^a^ The Ct values of influenza hemagglutinin (HA) subtyping real-time PCR that target H1 or H3 genes in pigs from different groups were compared by days using Kruskal-Wallis rank-sum test. The pairwise comparisons were conducted by Dunn’s test and Benjamin-Hochberg adjustment. The statistical significance (p < 0.05) is displayed by different superscripts (A, B).

**Table S3. Nonsynonymous variants in influenza hemagglutinin (HA) antigenic regions.**

| HA Type | Sample ID | Collection Date (dpc) ^a^ | Amino Acid Substitution ^b^ | Variant Frequency | Antigenic Region | Treatment Group |
| --- | --- | --- | --- | --- | --- | --- |
| H1 | 526 | 2 | N179D | 0.032 | Sa | SINGLE LAIV |
| H1 | 526 | 2 | L208I | 1.000 | Sb | SINGLE LAIV |
| H1 | 474 | 2 | L208I | 0.058 | Sb | NO VAC |
| H1 | 472 | 2 | L208I | 0.203 | Sb | H1 seeder |
| H1 | 508 | 2 | L208I | 0.127 | Sb | H1 seeder |
| H1 | 536 | 2 | K186R | 0.010 | Ca1 | H1 seeder |
| H1 | 536 | 2 | L208I | 0.112 | Sb | H1 seeder |
| H1 | 536 | 2 | S288L | 0.011 | Ca1 | H1 seeder |
| H1 | 614 | 3 | L208I | 1.000 | Sb | SINGLE LAIV |
| H1 | 562 | 3 | L208I | 0.012 | Sb | NO VAC |
| H1 | 560 | 3 | L208I | 0.130 | Sb | H1 seeder |
| H1 | 596 | 3 | L208I | 0.196 | Sb | H1 seeder |
| H1 | 624 | 3 | L208I | 0.020 | Sb | H1 seeder |
| H1 | 670 | 4 | K180R | 0.015 | Sa | NO VAC |
| H1 | 702 | 4 | L208I | 1.000 | Sb | SINGLE LAIV |
| H1 | 648 | 4 | L208I | 0.106 | Sb | H1 seeder |
| H1 | 684 | 4 | H155Y | 0.010 | Ca2 | H1 seeder |
| H1 | 684 | 4 | S207I | 0.029 | Sb | H1 seeder |
| H1 | 684 | 4 | L208I | 0.078 | Sb | H1 seeder |
| H1 | 790 | 5 | L208I | 0.997 | Sb | SINGLE LAIV |
| H1 | 772 | 5 | L208I | 0.456 | Sb | H1 seeder |
| H1 | 878 | 6 | L208I | 0.998 | Sb | SINGLE LAIV |
| H3 | 541 | 2 | R323G | 0.019 | C | SINGLE LAIV |
| H3 | 541 | 2 | A179S | 0.032 | B | SINGLE LAIV |
| H3 | 541 | 2 | E66G | 0.014 | C | SINGLE LAIV |
| H3 | 546 | 2 | A214T | 0.016 | B | H3 seeder |
| H3 | 562 | 3 | A202V | 0.021 | B | NO VAC |
| H3 | 633 | 3 | Q73R | 0.041 | E | SINGLE LAIV |
| H3 | 629 | 3 | S61N | 0.015 | C | SINGLE LAIV |
| H3 | 563 | 3 | R217K | 0.024 | D | H3 seeder |
| H3 | 593 | 3 | T203A | 0.010 | B | H3 seeder |
| H3 | 593 | 3 | S225N | 0.011 | D | H3 seeder |
| H3 | 687 | 4 | R254K | 0.044 | D | NO VAC |
| H3 | 809 | 5 | S162G | 0.012 | A | SINGLE LAIV |
| H3 | 897 | 6 | S281G | 0.018 | E | SINGLE LAIV |
| H3 | 897 | 6 | P243S | 0.038 | D | SINGLE LAIV |
| H3 | 899 | 6 | G145E | 0.011 | B | SINGLE LAIV |
| H3 | 897 | 6 | Q73R | 0.013 | E | SINGLE LAIV |

^a^ Collection date of the tested samples is shown as the number of days post contact (dpc) with the inoculated (seeder) pigs.

^b^ The amino acid sites were shown as H1 or H3 open reading frame numbering, including the signal peptide.
